# Systematic Ancient DNA Species Identification Fails to Find Late Holocene Domesticated Cattle in Southern Africa

**DOI:** 10.3390/biology9100316

**Published:** 2020-09-30

**Authors:** K. Ann Horsburgh, Anna L. Gosling

**Affiliations:** 1 Department of Anthropology and Department of Biology, Southern Methodist University, Dallas, TX 75028, USA; 2 School of Geography, Archaeology and Environmental Studies, University of the Witwatersrand, Wits 2050, South Africa; 3 Department of Anatomy, University of Otago, Dunedin 9016, New Zealand; anna.gosling@otago.ac.nz

**Keywords:** later stone age, South Africa, ancient DNA, cattle, bovid

## Abstract

**Simple Summary:**

Anthropologists reconstructing the spread of domesticated animals into new regions can often rely on the archaeological remains of those animals being readily distinguishable from the remains of wild species. In southern Africa there are several wild species with bones as resemble those of cattle. Non-morphological techniques must therefore be employed to identify cattle bones in southern African archaeological sites. We have used ancient DNA analyses to identify the species of bones from four southern African archaeological that we had hoped might be those of cattle. They were not. All the analyzed specimens came from wild species. Unfortunately this means that we must await further research to identify the earliest spread of domesticated animals to southern Africa.

**Abstract:**

Establishing robust temporal control of the arrival of domesticated stock and the associated husbandry skills and lifeways in Southern Africa remains frustrated by the osteological similarities between domestic stock and wild endemic fauna. We report the results of a systematic ancient DNA survey of appropriately sized bovid remains from Later Stone Age deposits in four South African archaeological sites. We show that none of the tested remains originated in domesticated cattle. The precise date of arrival of domestic cattle in the region awaits further study, although we also report new radiocarbon determinations which further refine the local chronology.

## 1. Introduction

For a few brief and satisfying years, it appeared that archaeologists had successfully constructed a secure chronology for the arrival of domesticated animals in Southern Africa [1]. With basic questions of timing controlled, research attention turned to more assessments of process. Arguments about the processes by which livestock husbandry became established in in Southern Africa has settled into two camps that are familiar from elsewhere: (1) that the development of animal husbandry was an indigenous phenomenon in which local foraging group adopted livestock and methods for their management from neighboring herding groups or, (2) herders moved in from the north and to some degree displaced but also lived alongside foraging groups.

Smith [2,3,4,5,6] has argued that the first herders in Southern Africa were migrants, bringing with them livestock, ceramics and Khoekhoe languages. He particularly argues that foragers have ideological impediments to storing food for the future, and a prevailing egalitarian ethic in which mean is shared, which preclude the private ownership necessary for successful livestock management. Sadr [7,8], by contrast, thinks that the transition to animal husbandry is relatively easy, and that foraging groups rapidly and without great cultural shift adopt livestock. In this he points to the ethnographically documented case of Kalahari Bushmen who began maintaining goats in the 1960s and 1970s, and who have nonetheless maintained the hunter-gatherer ideology of egalitarianism and the ethos of sharing so emphasized by Smith. He does see evidence of a migration of Khoekhoe speakers into Southern Africa, likely late in the first millennium AD, but only after herding groups, recently descending from local foraging groups, were established.

This tidy dichotomy has been recently complicated by Russell [9] who points out that there is no a priori reason to suppose that people, languages, domestic plants and animals, lithic manufacturing traditions, and ceramic styles should move into new regions as coherent and stable packages. Systematically integrating diverse archaeological datasets and taking seriously the ethnohistoric and ethnographic records [9,10] in recognition of the complexities of and variability in human behavior have begun to reveal more nuanced reconstructions of prehistoric lifeways in Southern Africa. 

Unfortunately, mounting evidence from ancient DNA [11,12,13,14,15,16,17,18,19] and mass spectrometry analyses [20] is revealing widespread misidentification of faunal remains. The first evidence was of wild fauna being assigned to domesticated taxa but more recent work has shown that the errors are bidirectional, with domestic fauna also being misidentified as wild taxa. Bovids in Southern Africa are abundant and taxonomically diverse [21], ranging in size from the tiny dikdik (*Madoqua kirki*) at only 4.5–5 kg to the eland, the largest of which weigh in excess of 900 kg [22]. Skeletal similarities between similarly sized bovids means that often specimens cannot be identified to species, or even genus. Commonly, therefore, archaeological remains of bovids are assigned to size classes, I–IV [22]. Size class I includes animals up to 23 kg including blue duiker (*Cephalophus monticola*), Cape grysbok (*Raphicerus melanotus*) and steenbok (*R. campestris*). Size class II animals range from 23 kg to 84 kg, including springbok (*Antidorcas marsupialis*), impala (*Aepyceros melampus*) and reedbuck (*Redunca arundinum*). Size class III ranges from 84 kg to 296 kg, including kudu (*Strepisiceros strepsiceros*), gemsbok (*Oryx gazella*) and nyala (*Tragelaphus agasi)*. Size class IV are those species larger than 296 kg including only the Cape buffalo (*Syncerus caffer*) and the eland (*Tragelaphus oryx*). Domestic cattle exhibit significant size range and can be found in both size classes III and IV [22]. While these categories are arbitrary, they are a useful analytical tool when identification to the level of species is not possible. We conducted genetic analyses of size class III-IV bovids from four archaeological sites in South Africa’s Western Cape Province, in the hope that some of them represented domestic cattle.

## 2. Methods and Materials

**Figure 1:**
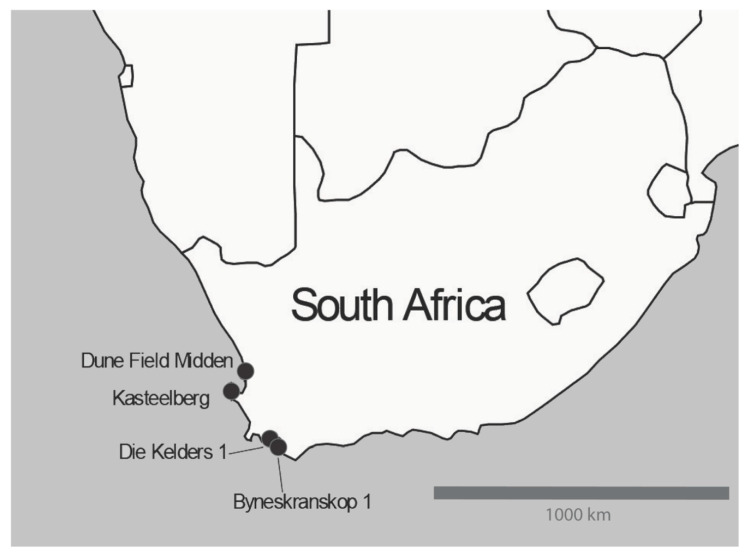
Site Information.

Twenty bovid specimens of size class III–IV, and therefore potentially cattle were selected from four archaeological sites in South Africa’s Western Cape Province. See Figure 1 for a map of the location of the sites, and below for a description of each site. All specimens were photographed before analysis. Digital copies of the photographs are held at the Iziko Museums of Cape town, at Heritage Western Cape, at the South African Heritage Resources Agency and with The Digital Archaeological Record (tDAR id: 457689).

### 2.1. Die Kelders 1 Cave

Die Kelders 1 Cave was first excavated between 1969 and 1972 [23] and again between 1992 and 1995 [24,25]. Strata include both Middle Stone Age (MSA) and Later Stone Age (LSA) deposits. The LSA deposits are in Layer 1 and are stratified in 12 subunits. Note that in some publications subunit 12 of Layer 1 is referred to as LSA Layer 12, and should not be confused with the MSA-bearing Layer 12. Ceramics are abundantly present in both subunit 12, the oldest of the LSA layers, and in subunit 2. Analyses of the sherds show that the ceramics recovered from these two subunits are significantly different from each other [24]. Unfortunately, too few sherds have been recovered from the intervening layers to reveal the rapidity of the change in ceramic technology. Sheep were recovered from Layer 1, subunit 2 (LSA Layer 2) and later subjected to ancient DNA analyses which reveals them to be very much like the other African sheep for which we have genetic data [26].

A single radiocarbon determination was has been reported from Layer 1, subunit 12 (LSA Layer 12), which calibrates to between 136 BC and AD 334 [GX-1688, 2 s.d., 23, SHCal13 radiocarbon curve, [27]. Four radiocarbon determinations have been reported from Layer 1, subunit 2 (LSA Layer 2), but Schweitzer, the original excavator reports that two of the dated samples (Gak3877 and 3878) were not recovered in situ and should therefore not be considered reliable indicators of the age of subunit 2 [28]. The other two determinations however (GX-1685 and GaK-3955) calibrate, at two standard deviations, to AD 412–857 and AD 215–756 [27,28].

Six potential cattle specimens from Layer 1, subunit 2 (LSA Layer 2), and one from Layer 1, subunit 12 (LSA Layer 12), were selected for ancient DNA analyses, and a specimen from Layer 1, subunit 2 (LSA Layer 2) was directly AMS dated. Morphological and stratigraphic details of each specimen can be found in Table 1.

### 2.2. Byneskranskop

Byneskranskop 1 is a stratified cave site with evidence of human occupation going back about 12,500 years [29]. Sheep remains have been recovered from Layer 1, from which there are three existing radiocarbon determinations. Two of the determinations (Pta-1964 and Pta-1866) calibrate, at two standard deviations, to AD 634–949 and AD 1324–1605 [27,29]. The third determination from Layer 1 (Pta-1865) calibrates, at two standard deviations, to AD 55–338. The ceramics and lithic assemblages from Layer 1 are somewhat sparse [30]. Faunal analyses show that the inhabitants continued to depend heavily on wild resources during the period in which sheep were first introduced to the site.

Six bovids of size class III–IV, all from Level 1, were selected for ancient DNA analysis. Four bovid specimens from Level 1, subunit O3 were directly radiocarbon dated by AMS. Morphological and stratigraphic details of each specimen can be found in Table 1.

### 2.3. Dunefield Midden

The Dunefield Midden is collection of LSA dune sites that suggest temporary occupation. The age distributions of the Cape fur seals (*Arctocephalus pusillus*) and dassies (rock hyrax, *Procavia capensis*) remains suggest further, that occupation was seasonal and likely confined to the winter months between March and October [31]. Faunal remains too, show evidence of significant shellfish predation with the presence of large shell middens comprised primarily of two species of limpets. The single class III–IV bovid specimen available for analysis came from the Dunefield Midden 1 site in the complex, which has a hearth with a calibrated date at two standard deviations of AD 692–992 (Pta-6734) [31]. Morphological and stratigraphic details of the specimen can be found in Table 1.

### 2.4. Kasteelberg

The 36 archaeological sites all called Kasteelberg (with various capital letter suffixes), take their name from the granite hill they surround. Of particular interest here are Kasteelberg A and Kasteelberg B. Only some of the Kasteelberg sites show evidence of sheep, but all show significant evidence of shellfish predation as well as the hunting of Cape fur seals (*Arctocephalus pusillus*) and wild terrestrial bovids [32,33,34,35,36]. Like the Dunefield Midden sites, Kasteelberg seems to have been occupied seasonally. At nine months of age Cape fur seals are weaned and no longer sheltered by maternal care. Many seals of this age wash up on the beaches exhausted or dead providing rich resources for easy harvest. The majority of the fur seal remains from Kasteelberg were approximately nine months old, meaning both that the residents of the sites were in residence to take advantage of the bounty and that the occupation period was likely between mid-August and early October, when the seasonally breeding seals are nine months old [32]. This is consistent too, with Smith’s [36] argument that these sites were likely occupied, at least in part, to facilitate that exploitation of marine mammal fat available from natural strandings on the nearby coastline.

The stratigraphies of the Kasteelberg sites are notably complex [35], but most deposits likely date between about AD 1000 and AD 1200 [33,34,35,36]. Two bovid specimens from Kasteelberg A and four from Kasteelberg B were analysed for ancient DNA, and one bovid specimen from Kasteelberg B was submitted for direct AMS dating. Morphological and stratigraphic details of the specimen can be found in Table 1.

### 2.5. Ancient DNA Laboratory Analyses

Extractions of ancient DNA and preparation of Illumina sequencing libraries was undertaken in the ancient DNA laboratories of the SMU Molecular Anthropology Laboratories and of the Department of Anthropology at the University of Utah using methods described previously [13]. In brief, DNA was extracted from bone or tooth fragments weighing between 520 mg and 1.86 g using a silica and guanidinium thiocyanate protocol [37,38]. A negative control was processed in parallel with every group of five samples. DNA sequencing libraries for the Illumina Hi-Seq platform were constructed directly from the ancient DNA extracts using barcoded P7-adapters following Meyer and Kircher [39]. The number of PCR cycles to amplification plateau was determined with quantitative PCR using Bio-Rad’s CFX96 Touch Real-Time PCR Detection System and SYBR Green dye (Applied Biosystems, Foster City, CA, USA). Amplified libraries were visualized with ethidium bromide on 2% agarose gels. Libraries were then immortalized by PCR amplification using ABI’s AmpliTaq Gold (1× AmpliTaq PCR Buffer II, 2.5mM MgCl_2_, 1mM dNTPs, 0.2 μM of each extension primer and 3.75 units of AmpliTaq Gold). Immortalized libraries were purified over silica columns (Zymo Clean and Concentrator, Zymo Research, Irvine, USA) according to manufacturer’s instructions modified only by a second wash with the DNA Wash Buffer and 5 min incubation with 0.1× TE buffer instead of the prescribed 30 s incubation with the provided DNA Elution Buffer.

DNA sequencing libraries were enriched for the mitochondrial genome using MyBait probes designed for cattle (MYcroarray, Ann Arbor, MI, USA) following the manufacturer’s protocol for low quantity and low quality targets. Enriched sequencing libraries were quantified by qPCR as above, pooled in equimolar ratios and sequenced with 100PE reads on the Illumina HiSeq 4000 platform at the University of Chicago Genomic Facility.

Raw fastq files were processed using AdapterRemoval2 (v. 2.1.7), which removed sequencing adapters and merged paired end fragments, which overlapped by at least 11 base-pairs. Reads were also processed to remove stretchs of Ns, bases that had a low quality score (<30), and short reads (<25). The collapsed reads output by this process were aligned used the Burrow Wheeler Alignment (BWA v. 0.7.17) against the mitochondrial reference genome for domesticated cattle (*Bos taurus*, Genbank ID V000654.1) with recommended ancient DNA settings; the Aln function was used, seeding disabled (−l 1024), the number of gap opens was set to 2 (−o 2), and the maximum edit distance was set to 0.03 (−n 0.03).

PCR duplicates were removed using Samtools (v. 1.9) markdup −r. To confirm the authenticity of the ancient DNA, the program mapDamage (v. 2.0.2) which identified characteristic aDNA damage patterns, was used with the ‘-rescale’ option to lower the quality score of likely damaged sites. The plots of damage patterns were created for each sample and examined for the characteristic C to T and G to A transitions at the end of the reads (see Supplementary Figure S1).

A coverage plot was generated using the ggplot2 package for the R programming language. This was examined to assess mean coverage across the mitochondrial genome, as well as percent total coverage. Because the individuals in question were not in fact *Bos taurus*, these coverage plots appeared patchy and incomplete. As such, an alternate approach was taken.

A composite reference file containing individual FASTA sequences of the mitochondrial genomes of a number of different potential other candidate species was created. These included other ungulates of approximately a similar size to cattle, whose bony elements might be physically similar. This composite reference file included mitochondrial genomes for cattle (*Bos taurus*, Genbank ID V000654.1), Cape buffalo (*Syncerus caffer*, Genbank ID NC_020617.1), eland (*Tragelaphus oryx*, Genbank ID NC_020750.1), gemsbok (*Oryx gazella*, Genbank ID: JN869312.1), and extinct Blue antelope (*Hippotragus leucophaeus*, Genbank ID NC_035309.1).

Alignment against this composite reference sequence and the subsequent steps were conducted as previously described. The idxstats command in Samtools (v. 1.9) was used to output the relative number of reads mapping to each reference genome included in the composite reference file. The species with the most reads assigned to it was assumed to be the species of origin for the bone from which DNA was extracted. Maximum likelihood trees were calculated to test the possibility that the archaeological bones represented species for which mitochondrial genomes are not publicly available. This was done using the SMS tool in PhyML 3.0 [40] to determine the best model for constructing the ML tree. Using the AIC selection criterion, SMS determined that the most parsimonious tree used a GTR + G substation model. As can be seen in Figure 1 and Supplementary Figure S4, the sequenced specimens fall within the range of variation expected for these species. As previous, coverage plots were generated using a ggplot2.

Using the Genome Analysis Toolkit’s (GATK v. 3.5) Haplotype Caller, the reads that mapped to the determined species’ reference genome were extracted, using settings specific for haploid genomes. This file was filtered for mapping quality (<20), and bases supported by fewer than 3 reads. This approach was justified under the assumption that the above quality control preferentially reduces the quality of the damaged positions in the sequencing reads, which would lead to a reduction in the probability that the damaged position would be called downstream. A consensus sequence containing indels (insertions and/or deletions) was created. Sites that were supported by fewer than 3 reads were changed to Ns. BLAST searches were used across the NCBI data database to confirm the species identifications. PhyML 3.0 [41] was used to map the generated consensus sequences against mitochondrial genomes from other publicly available data for these species.

## 3. Results

DNA was successfully recovered and sequenced from 13 of the analyzed 20 specimens. Initial alignment of the DNA libraries against the *Bos taurus* reference sequence showed patchy coverage (see Supplemental Figure S2), principally because the specimens for which we could recover DNA from did not belong to this species. The DNA sequences recovered proved to be of wild origin. We report here the species identified, and new radiocarbon determinations for three of the archaeological sites. See Table 2 for species assignments and details of the new radiocarbon determinations. The raw data through NCBI’s Sequence Read Archive (SRA, project number PRJNA655802). The number of reads mapping to each possible species are included in Supplementary Table S1, and coverage plots in Supplementary Figure S3. Mean mitochondrial coverage and percent coverage for the species determined most likely in each case are included in Table 2.

All five specimens from Die Kelders 1 that provided analyzable DNA were excavated from Layer 2. The specimens are all *Taurotragus oryx* (eland). The new radiocarbon determination from a specimen from Layer 1, subunit 2, C3 (LSA Layer 2) calibrates [42] to AD 688–968. Three specimens from Byneskranskop 1 were successfully sequenced, and identified as *Syncerus caffer* (the Cape buffalo). We report here four new radiocarbon determinations from specimens excavated from Level 1, O32. The two the dated specimens did not yield DNA allowing an accurate species identification calibrate to 199 BC–AD 25 and AD 122–341. The two dated specimens identified as Cape buffalo calibrate to AD 69–321 and AD 245–418. The single specimen from the Dunefield Midden is *Oryx gazella* (gemsbok). The single specimen from Kasteelberg A that provided DNA is from a *Taurotragus orxy* (eland), and the two from Kasteelberg B are *Taurotragus orxy* (eland) and *Syncerus caffer* (the Cape buffalo). The Cape buffalo from Kasteelberg B has a calibrated date of AD 989–1150.

## 4. Discussion

Southern African archaeological sites that span a period of a few hundred years, approximately two thousand years ago, document an economic shift from a system based entirely on the acquisition of wild foodstuffs, to one involving food production. The archaeological and ethnohistoric records detail a pastoral economy dependent on both the procurement of wild foods and the husbandry of domesticated sheep (*Ovis aries*), cattle (*Bos taurus*) and dogs (*Canis familiaris*). The ethnohistoric record identifies these groups as Khoikhoi; however, many aspects of both the origins and the practice of pastoralism in Southern Africa remains obscured by time.

The radiocarbon dates obtained in this study are largely consistent with previously reported occupation periods, but offer some refinement. The new determination from Layer 1, subunit 2, C3 (LSA Layer 2) at Die Kelders 1 extends the period of likely occupation to AD 968. Conversely, the new determinations from Byneskranskop pushes the dating of Level 1 back more than 200 years. The new date from Kasteelberg B is consistent with previously reported dating of the site.

Currently, the best evidence for the transition to food production in Southern African sheep remains directly radiocarbon dated to ~2000 years ago [28,43,44,45,46]. Cattle and dogs do not appear in the record in such abundance, and when they are present their osteological similarities to other Southern African bovids in size class III–IV has made their identification uncertain, and therefore, the timing of their arrival equivocal. While ancient DNA data are sometimes over-interpreted in archaeological contexts, DNA sequence particularly well suited for accurate species identification [47]. Unfortunately, this study has identified only wild species among specimens that we reasonably hoped might represent domestic stock. It is clear, from the faunal assemblages at Die Kelders 1 [25] and Byneskranskop [29], for example, that wild resources continued to be important food resources after the introduction of sheep to the Western Cape Province of South Africa. The determination that the specimens analyzed here all represent wild specimens does not, therefore, imply an absence of domestic forms in the assemblages of these sites. While the genetic identifications of archaeological fauna remain contentious in Southern Africa, the results obtained here further support the conclusion that caution is warranted in making morphological identifications of fragmentary specimens, and that the conservative identification of the specimens analyzed here to size class, and not to species, was appropriate. The only genetically identified cattle specimens that have been directly dated from Southern Africa are a horn corn from KN2005/041 in Namaqualand which calibrates to AD 421–559 [17] and a distal fragment of a first phalanx from Sehonghong in Lesotho which calibrates to AD 814–976 [14]. Secure identification of the first cattle in the Western Cape Province of South Africa must await further research.

## 5. Conclusions

Analyses of ancient DNA are valuable in their ability to accurately identify archaeological bones to the species level. We generated mitochondrial genome sequences from specimens excavated from four southern African archaeological sites. These data showed that none of the tested specimens originated in domesticate cattle, but were all from wild species. Without further research we cannot therefore accurately date the arrival of cattle in southern Africa.

## Figures and Tables

**Table 1:** Morphological and stratigraphic details of analyzed specimens.

Sample ID	Site	Element	Morphology	Provenience
DK1_62	Die Kelders 1	R lunate	Complete	Layer 2, D2
DK1_64	Die Kelders 1	R scaphoid	Complete	Layer 2, D2
DK1_65	Die Kelders 1	R magnum	Complete	Layer 2, C3
DK1_66	Die Kelders 1	R metacarpal	Complete	Layer 2, C3
DK1_67	Die Kelders 1	L cuneiform	Complete	Layer 2b, B3
DK1_68	Die Kelders 1	R 1st phalanx	Complete	Layer 2, A99
DK1_69	Die Kelders 1	R 1st phalanx	Distal third	Layer 1, subunit 12
BNK1_01	Byneskranskop	L mandible	dp2 dp3 dp4	Level 1, O32
BNK1_02	Byneskranskop	R P3	Slightly worn	Level 1, O32
BNK1_03	Byneskranskop	L 1st phalanx	Proximal fragment	Level 1, O32
BNK1_04	Byneskranskop	Rib	fragment	Level 1, O32
BNK1_05	Byneskranskop	L tibia	Proximal fragment	Level 1, O32
BNK1_06	Byneskranskop	R 1st phalanx	Distal fragment	Level 1A, P32
DFM_07	Dunefield Midden	R dp3	Complete	JAC15 #7
KBA_20	Kasteelberg A	1st phalanx	Epiphyses fused	9a’ 0–10
KBA_21	Kasteelberg A	L 2nd phalanx	Complete	13b’ 30–40
KBB_22	Kasteelberg B	R metatarsal	Proximal fragment	CS J4
KBB_23	Kasteelberg B	Metacarpal	Distal fragment	BSL2 H5
KBB_24	Kasteelberg B	L scaphoid	Complete	ONWIS F6
KBB_25	Kasteelberg B	L phalanx	Proximal fragment	POG 17

**Table 2:** Specimen Information.

Sample ID	Site	Species	C14	Provenience	% mtDNA Coverage	Mean mtDNA Coverage
DK1_62	Die Kelders 1	Eland		Layer 2, D2	99.6%	129.9
DK1_64	Die Kelders 1	Eland		Layer 2, D2	99.5%	172.1
DK1_65	Die Kelders 1	-	(Beta-232592–1240 +/− 40)	Layer 2, C3		
DK1_66	Die Kelders 1	Eland		Layer 2, C3	98.3%	98.3
DK1_67	Die Kelders 1	Eland		Layer 2b, B3	91.2%	4.2
DK1_68	Die Kelders 1	Eland		Layer 2, A99	99.3%	191.5
DK1_69	Die Kelders 1	-		Layer 1, subunit 12		
BNK1_01	Byneskranskop	Buffalo		Level 1, O32	99.0%	19.6
BNK1_02	Byneskranskop	-	Beta-232588–1840 +/− 40	Level 1, O32		
BNK1_03	Byneskranskop	-	Beta-232589–2100 +/− 40	Level 1, O32		
BNK1_04	Byneskranskop	Buffalo	Beta-232590–1740 +/− 40	Level 1, O32	99.0%	9.2
BNK1_05	Byneskranskop	Buffalo	Beta-232591–1880 +/− 40	Level 1, O32	86.6%	3.1
BNK1_06	Byneskranskop	Eland		Level 1A, P32	99.6%	103
DFM_07	Dunefield Midden	Gemsbok		JAC15 #7	94.0%	131.5
KBA_20	Kasteelberg A	Eland		9a’ 0–10	98.9%	25
KBA_21	Kasteelberg A	-		13b’ 30–40		
KBB_22	Kasteelberg B	Buffalo	Beta-232593–1040 +/− 40	CS J4	99.7%	137.4
KBB_23	Kasteelberg B	-		BSL2 H5		
KBB_24	Kasteelberg B	-		ONWIS F6		
KBB_25	Kasteelberg B	Eland		POG 17	97.2%	10.3

## Supplementary Materials

The following are available online at https://www.mdpi.com/2079-7737/9/10/316/s1, Figure S1: Typical aDNA Damage, Figure S2: Coverage Plots Against *Bos taurus*, Figure S3: Coverage Plots Against Determined Species, Figure S4: Maximum Likelihood Trees, Table S1: Reads mapped to species.
