# Supplementary material for: Systematic Ancient DNA Species Identification Fails to Find Late Holocene Domesticated Cattle in Southern Africa
Source: Biology (Basel). 2020 Sep 30;9(10):316. doi: 10.3390/biology9100316 (PMC7600501; doi:10.3390/biology9100316)
Supplement: Supplementary file 1 [file biology-09-00316-s001.zip › biology-912270-supplementary-figs.docx]

**Supplementary Figures**

**Figure S1: Example of typical aDNA damage, showing C>T damage at the 5’ end, and G>A damage at the 3’ end of the DNA fragments**
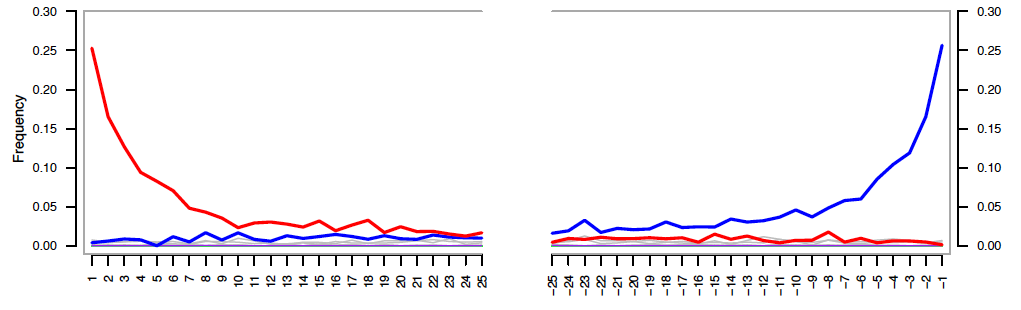


Figure 1: Nucleotide mis-incorporation plot for sample BNK1_01 as output by MapDamage

**Figure S2: Coverage plots showing initial mapping against *Bos taurus* mitochondrial genome**

**2.1 BNK01_01**

71.5% coverage, mean 4.2x

**
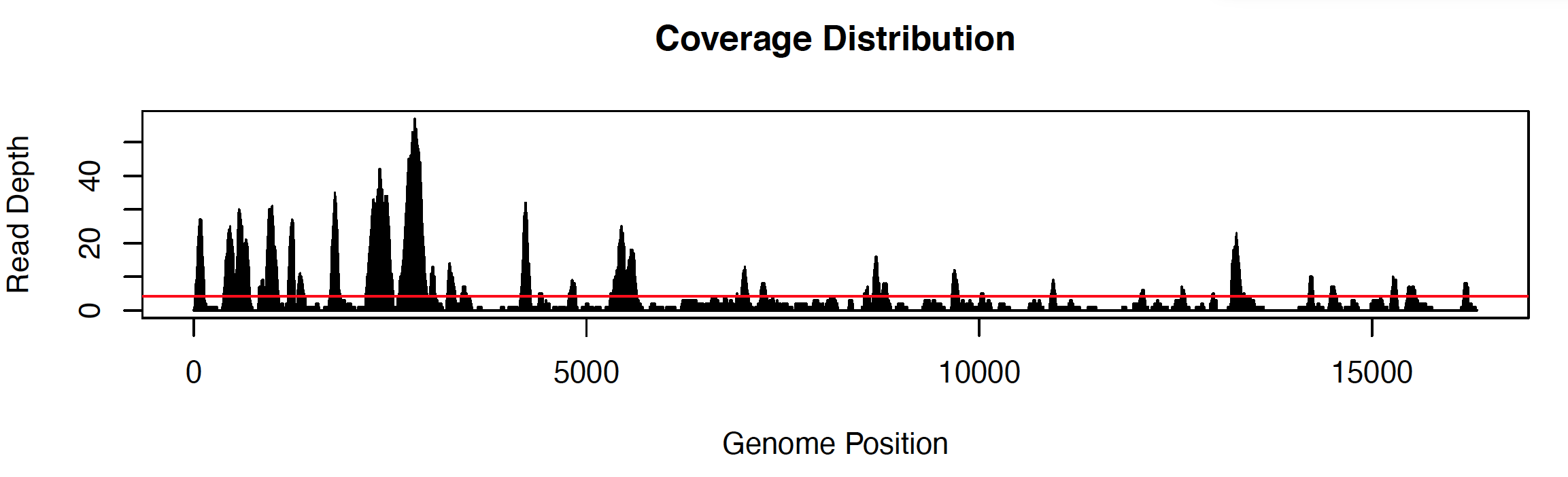
**

**2.2 BNK01_04**

74.3% coverage, mean 2.4x

**
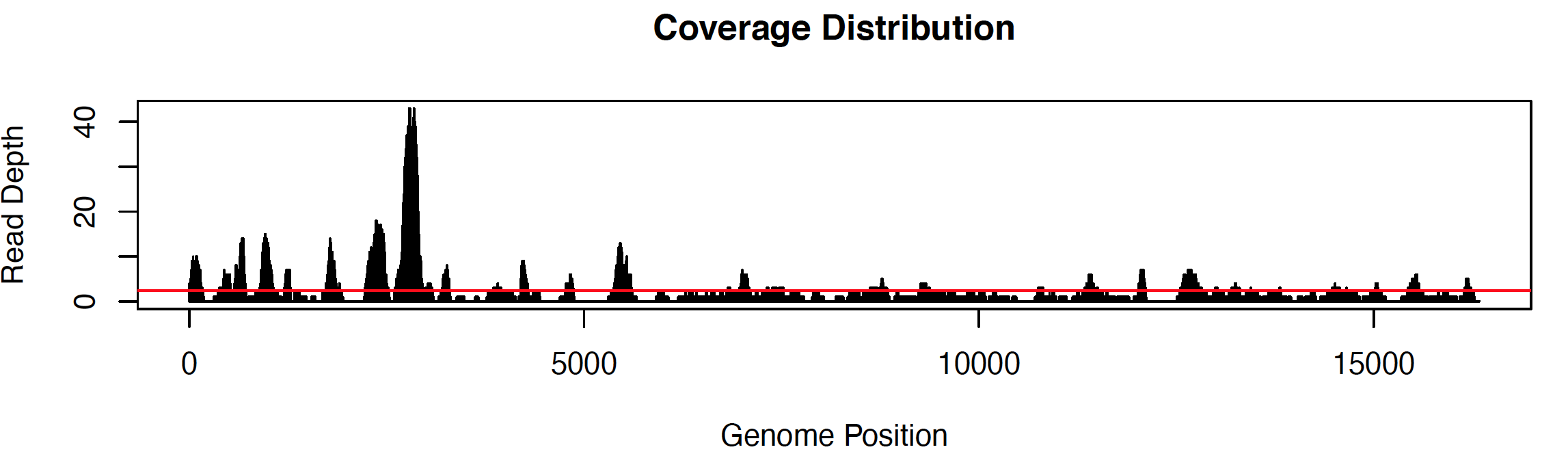
**

**2.3 BNK01_05**

53.2% coverage, mean 2.6x

**
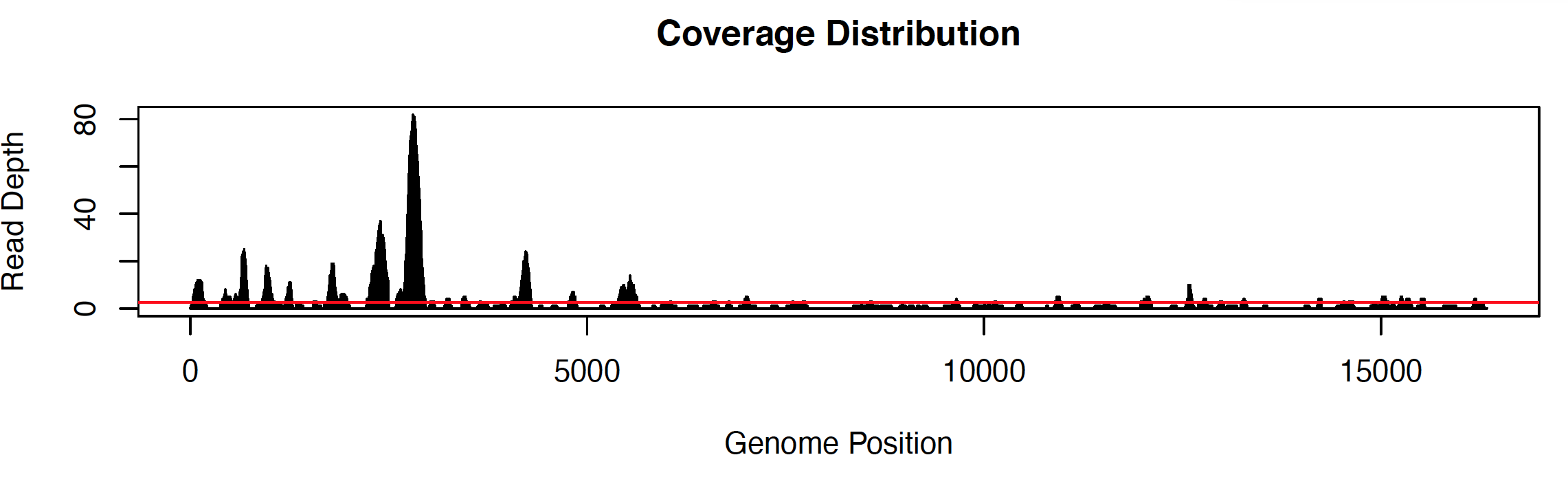
**

**2.4 BNK01_06**

89.4% coverage, mean 9.4x


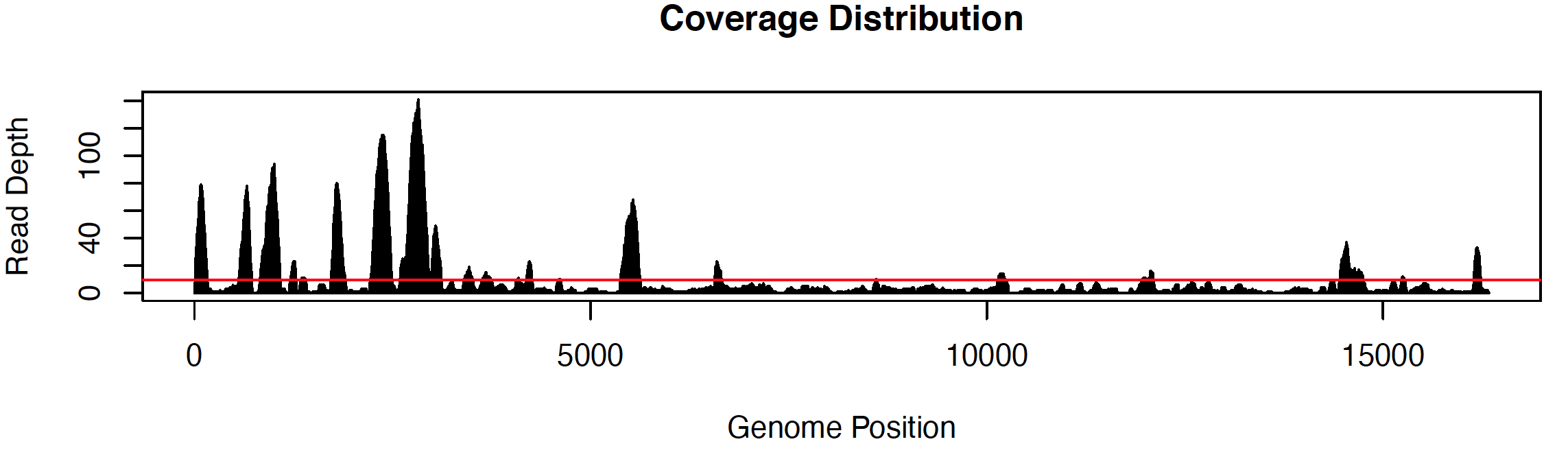


**2.5 DFM_07**

71.7% coverage, mean 8.2x


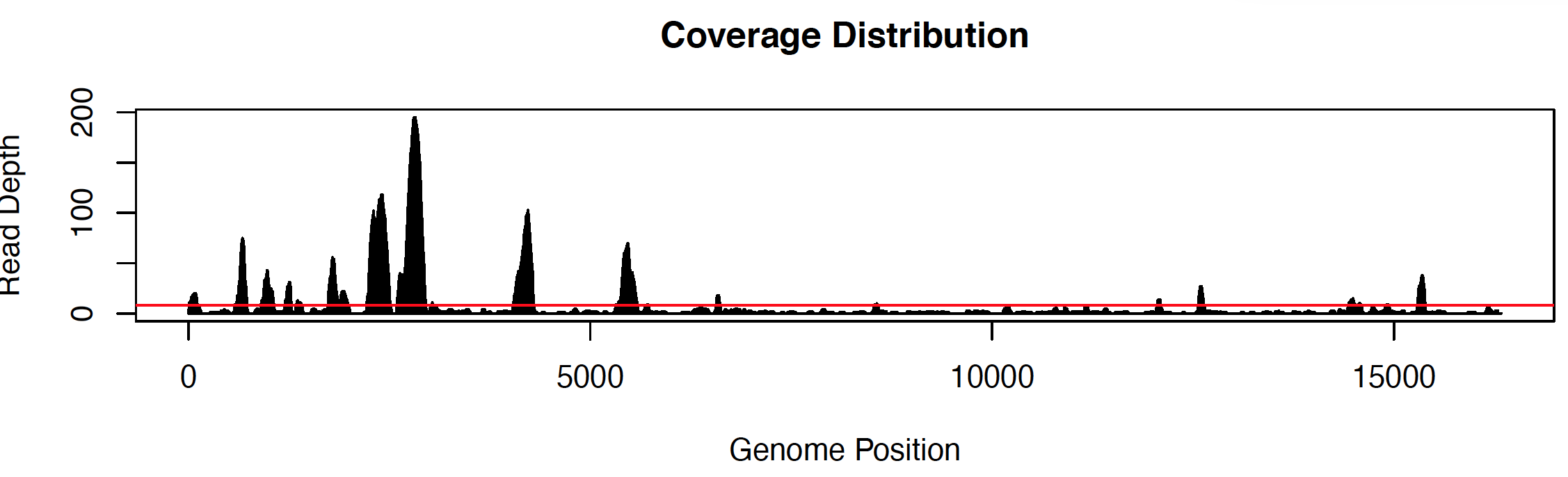


**2.6 DK1_62**

46.2% coverage, mean 15.7x


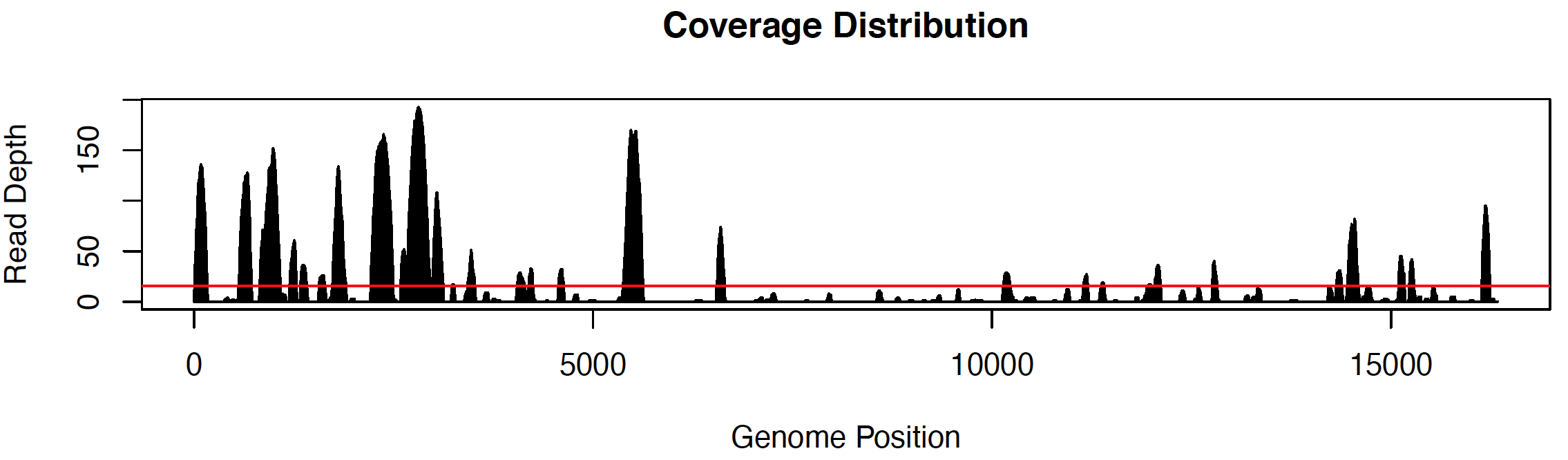


**2.7 DK1_64**

91% coverage, mean 19.6x


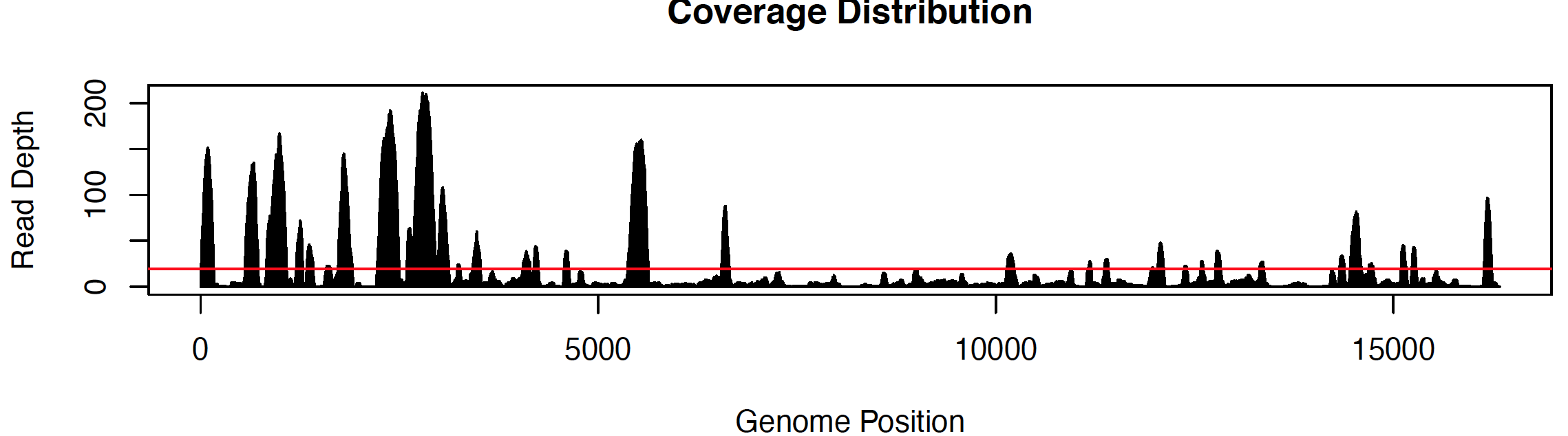


**2.8 DK1_66**

45.1% coverage, mean 10.7x

**
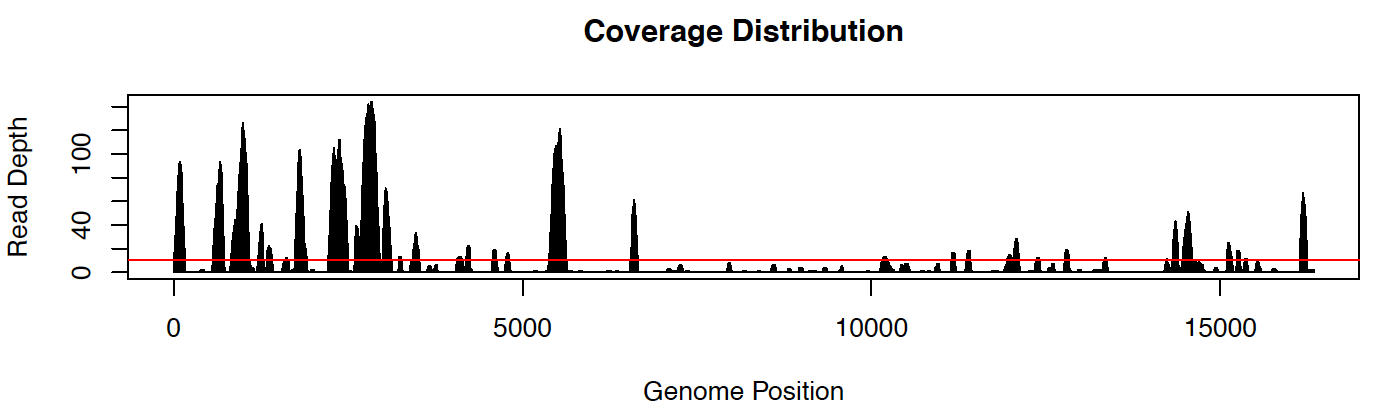
**

**2.9 DK1_67**

61.7% coverage, mean 1.7x
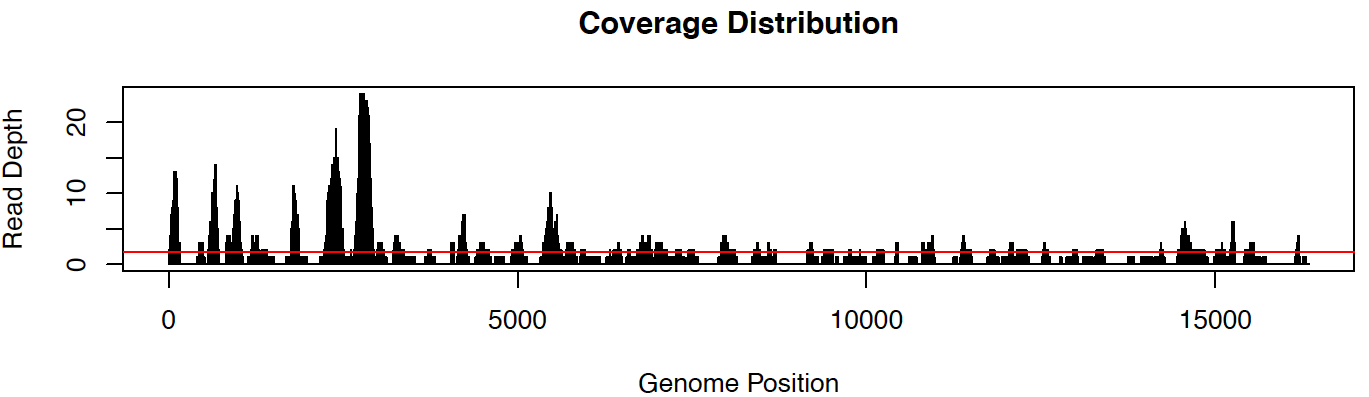


**2.10 DK1_68**

55.1% coverage, mean 28.3x

**
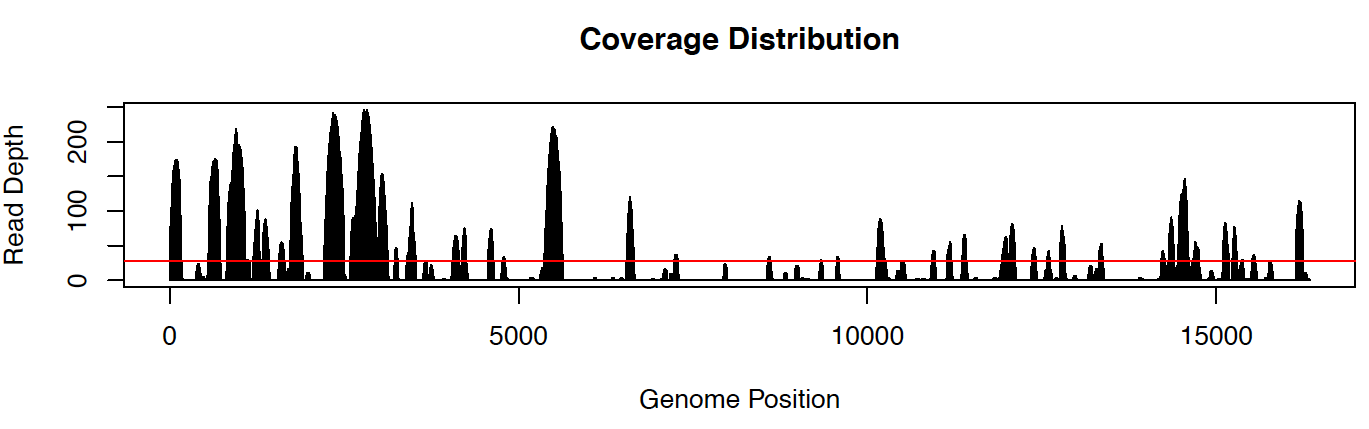
**

**2.11 KBA_20**

31.1% coverage, mean 2.6x

**
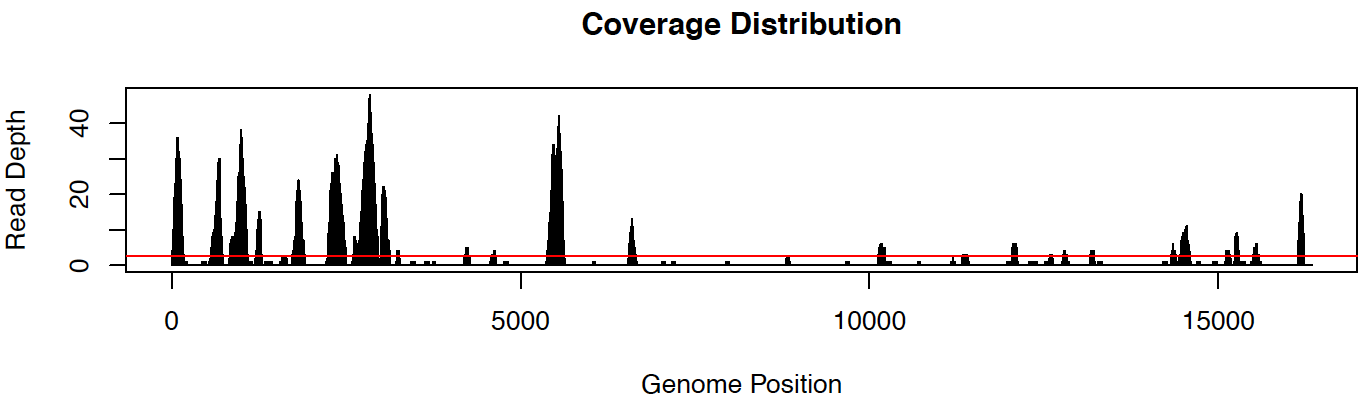
**

**2.12 KBB_22**

82.8% coverage, mean 12.9x

**
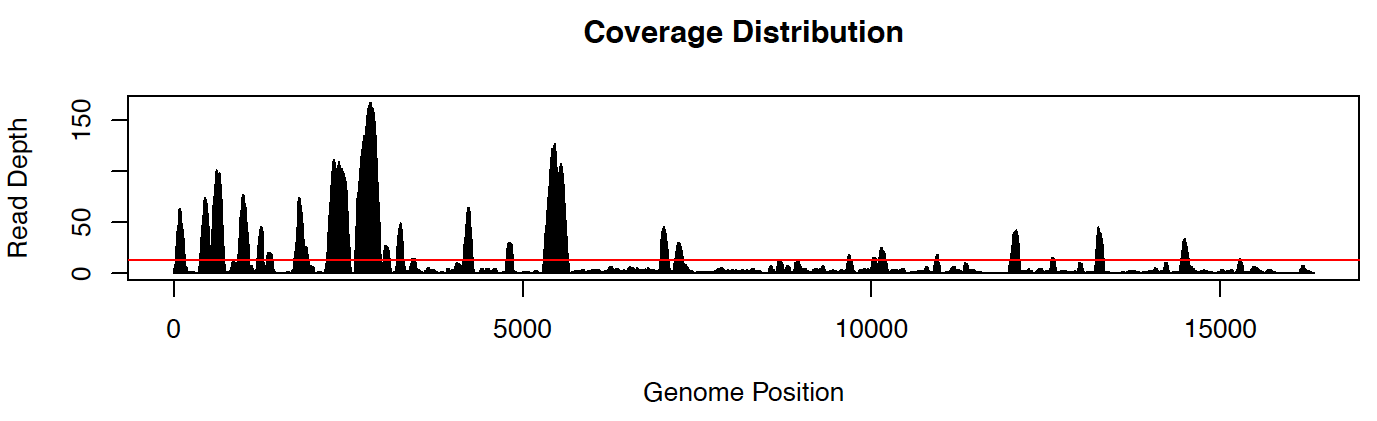
**

**2.13 KBB_25**

33.8% coverage, mean 1.1x

**
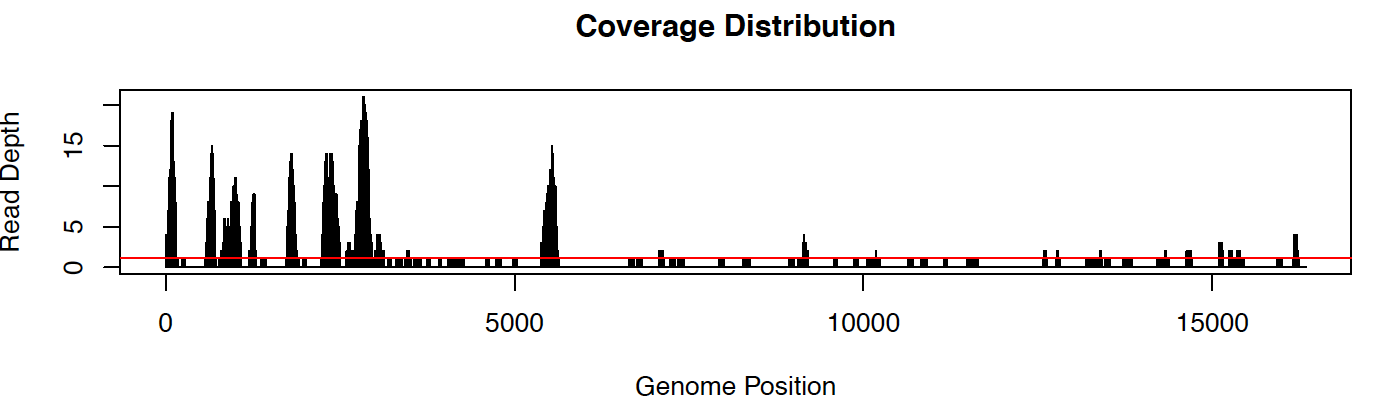
**

**Figure S3: Coverage plots showing mapping against determined species mitochondrial genome**

**3.1 BNK01_01 (Buffalo)**

99% coverage, mean 19.6x


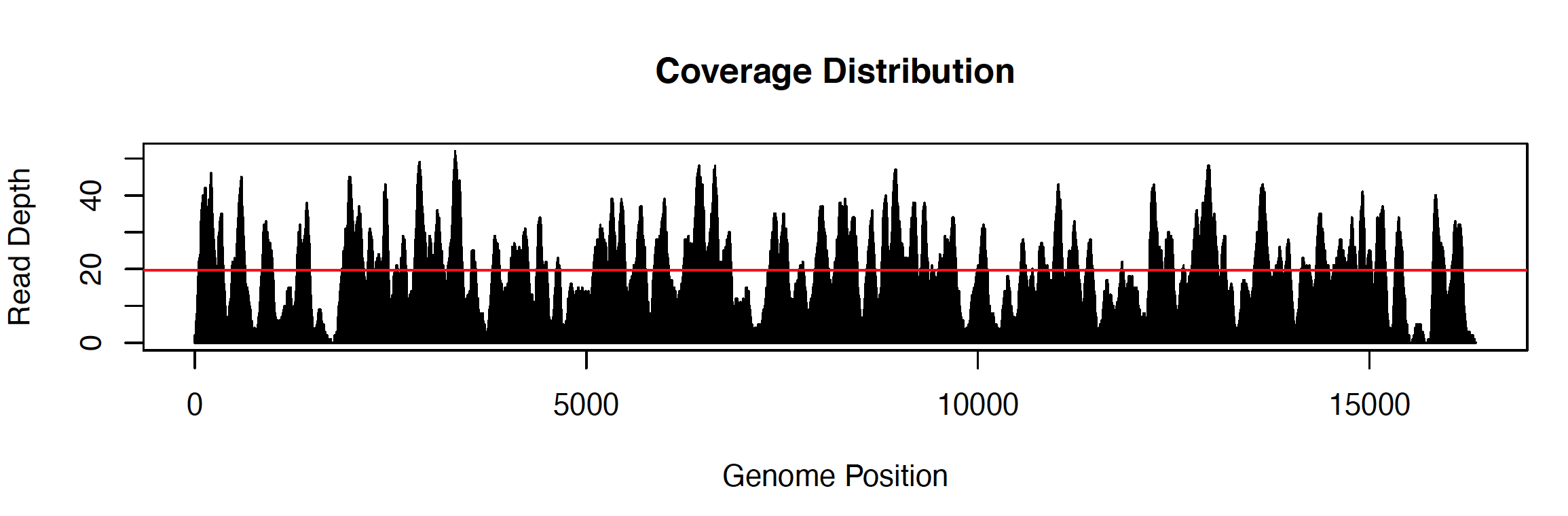


**3.2 BNK01_04 (Buffalo)**

99% coverage, mean 9.2x

**
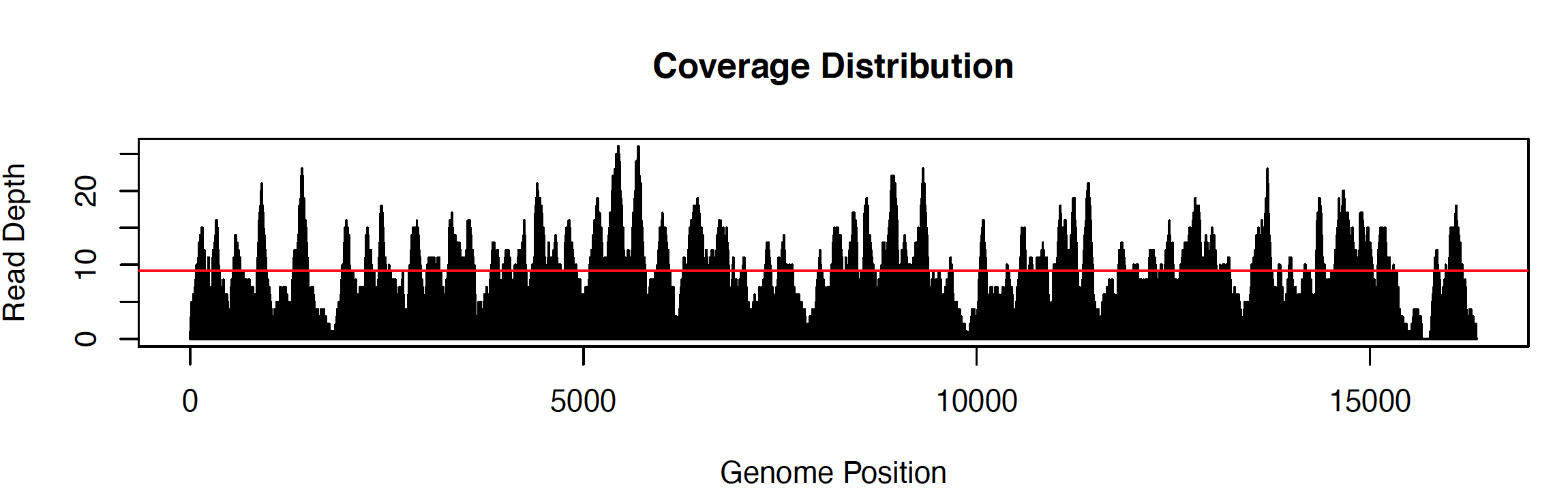
**

**3.3 BNK01_05 (Buffalo)**

88.6% coverage, mean 3.1x


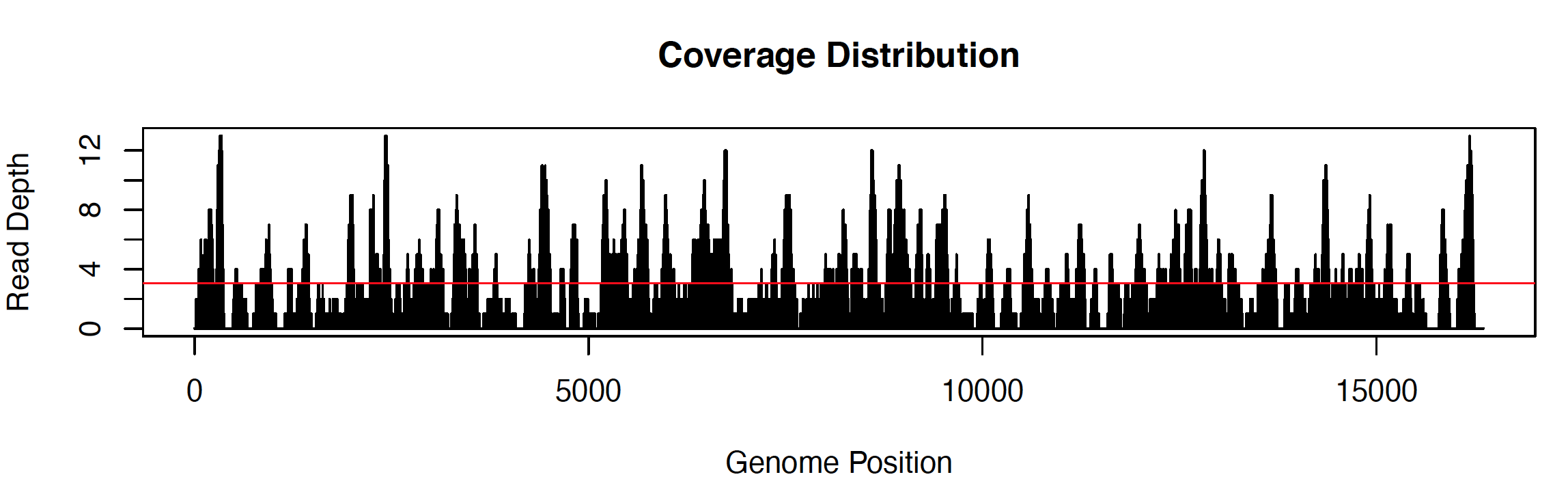


**3.4 BNK01_06 (Eland)**

99.6% coverage, 103.6x


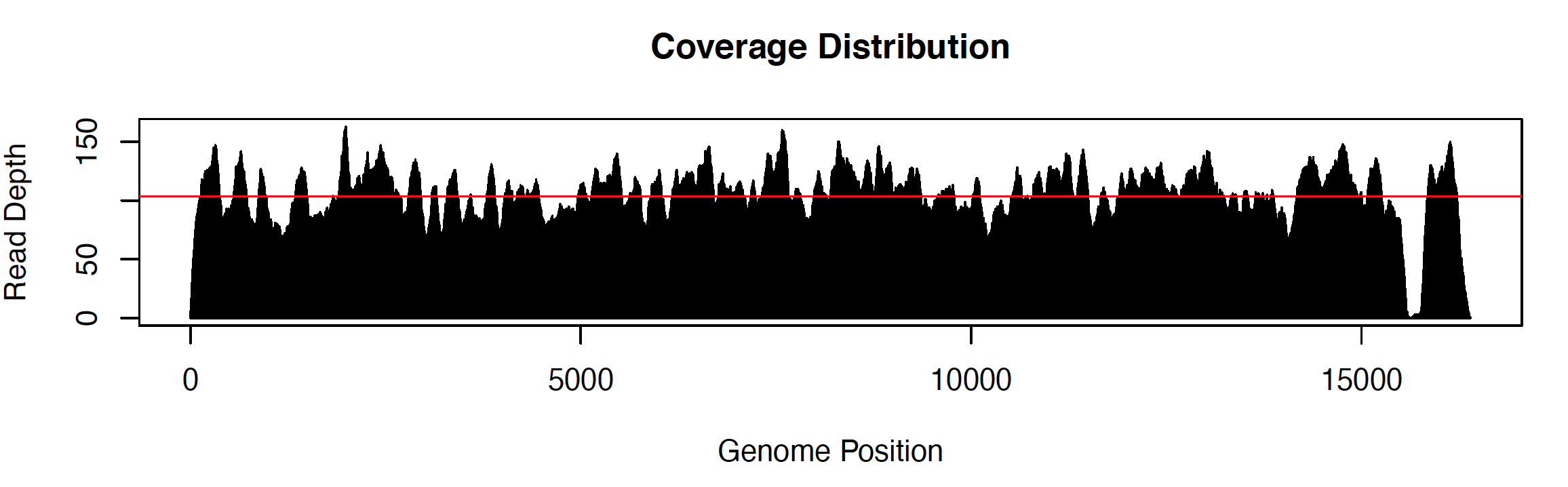


**3.5 DFM_07 (Gemsbok)**

94% coverage, mean 131.5x

**
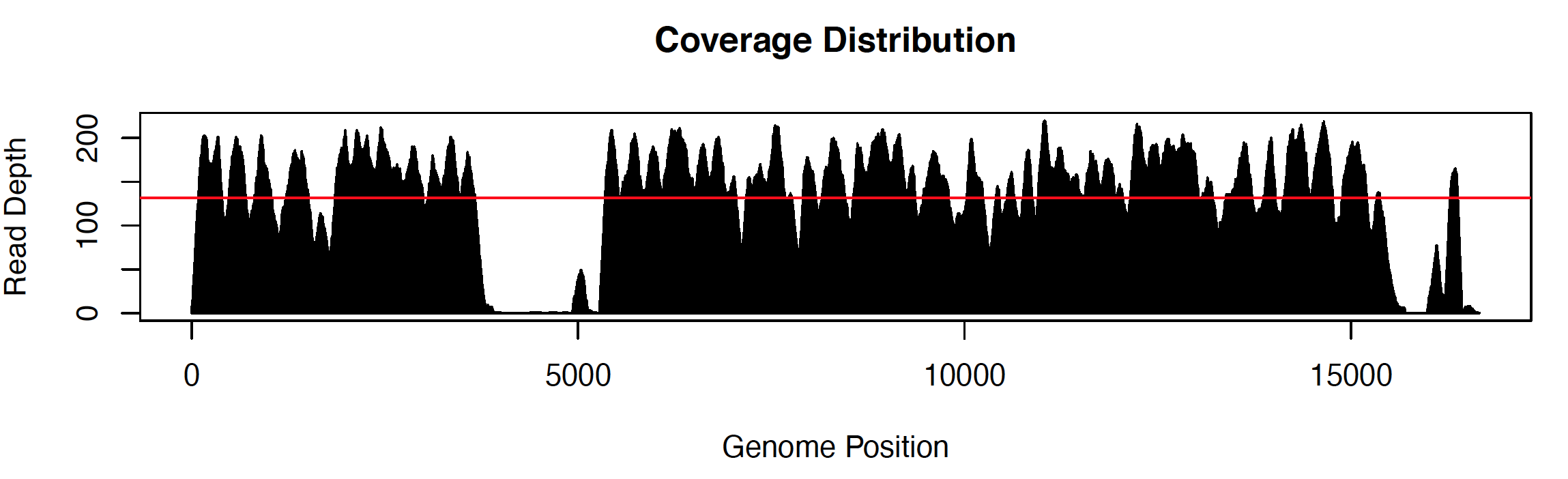
**

**3.6 DK1_62 (Eland)**

99.6% coverage, mean 129.9x


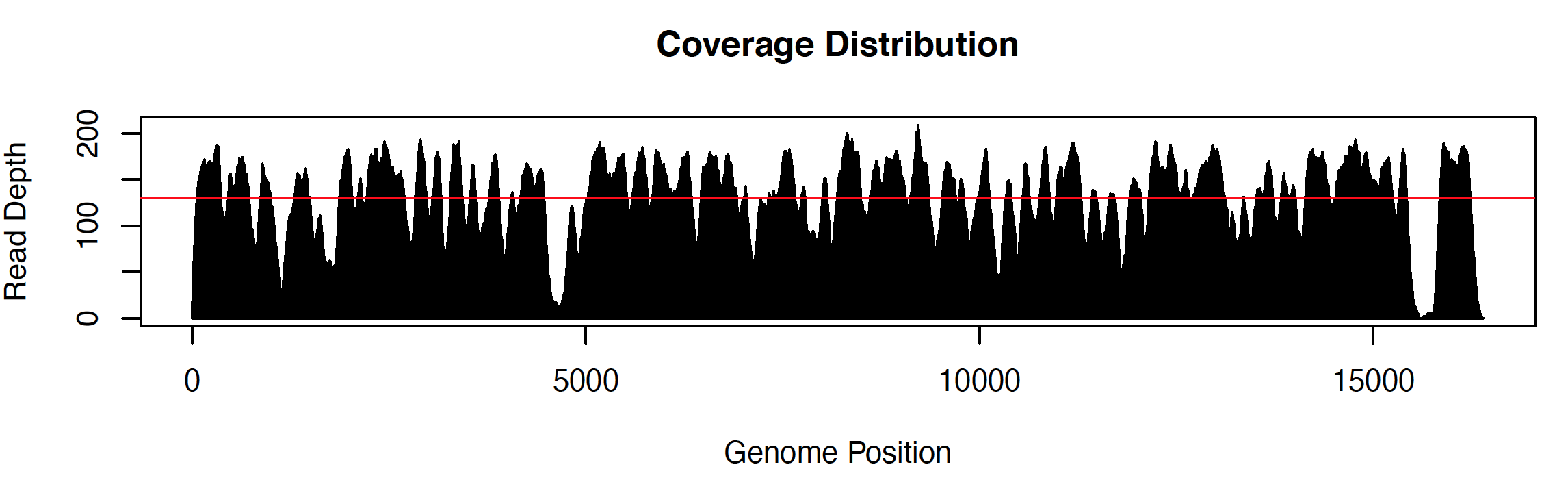


**3.7 DK1_64 (Eland)**

99.5% coverage, mean 172.1x

**
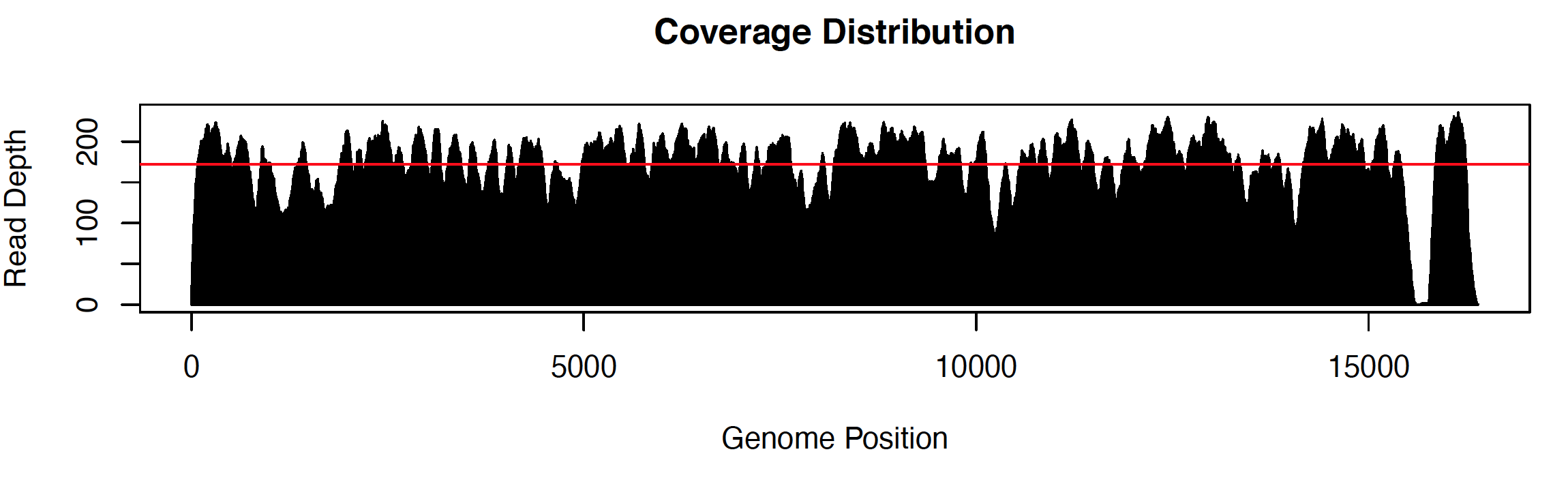
**

**3.8 DK1_66 (Eland)**

98.8% coverage, mean 98.3x


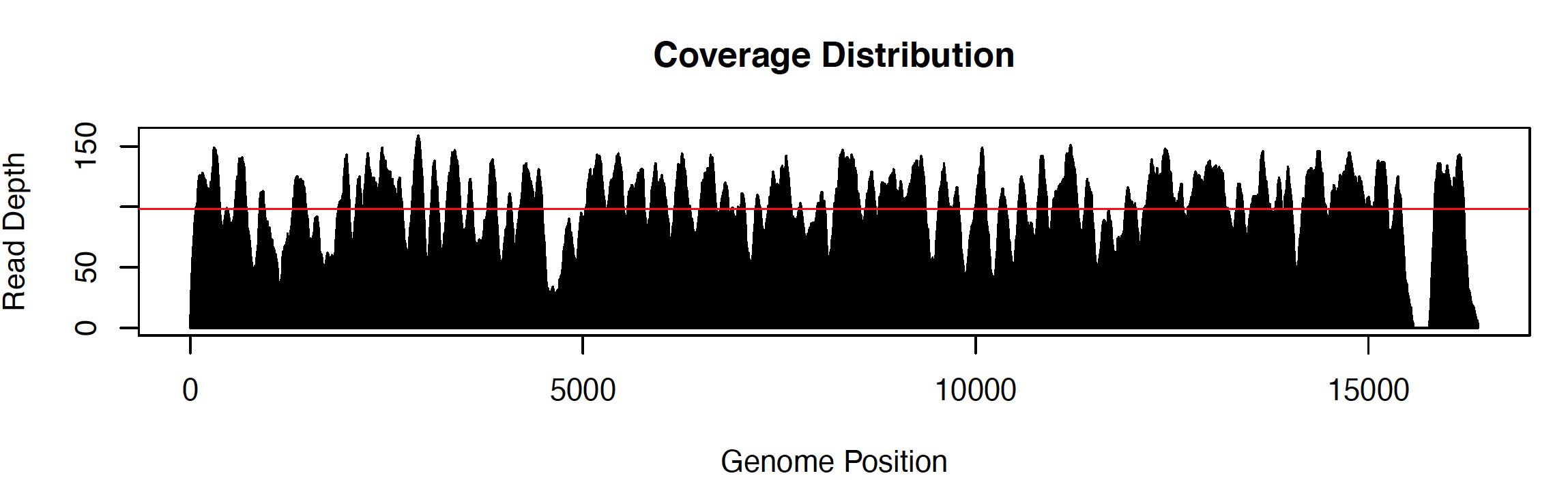


**3.9 DK1_67**

91.2% coverage, mean 4.2x


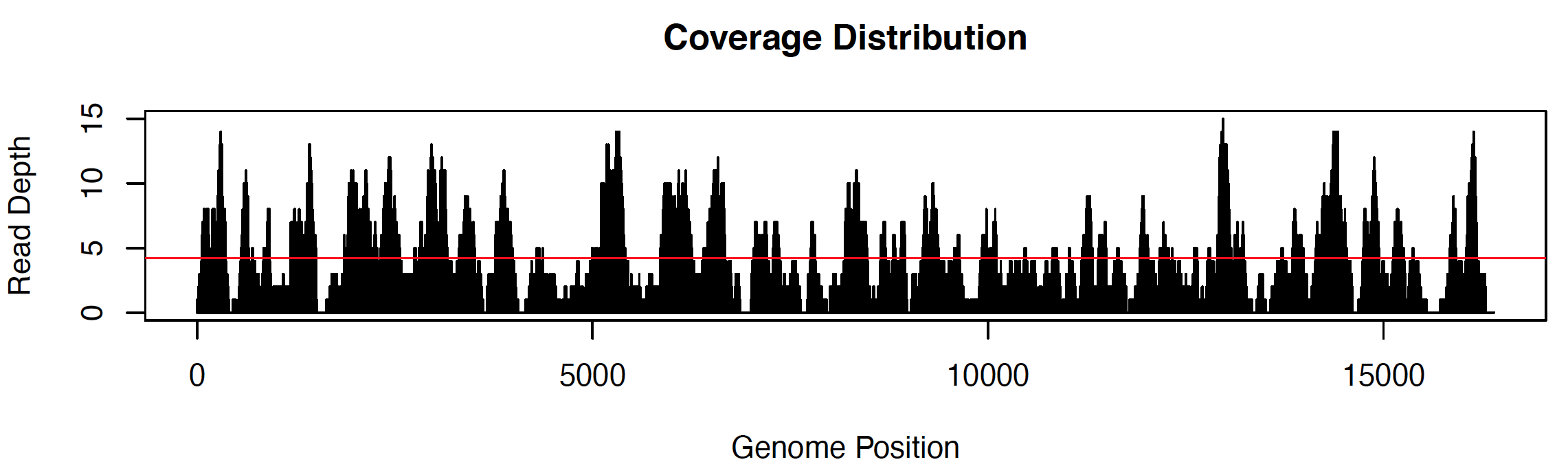


**3.10 DK1_68**

99.3% coverage, mean 191.5x

**
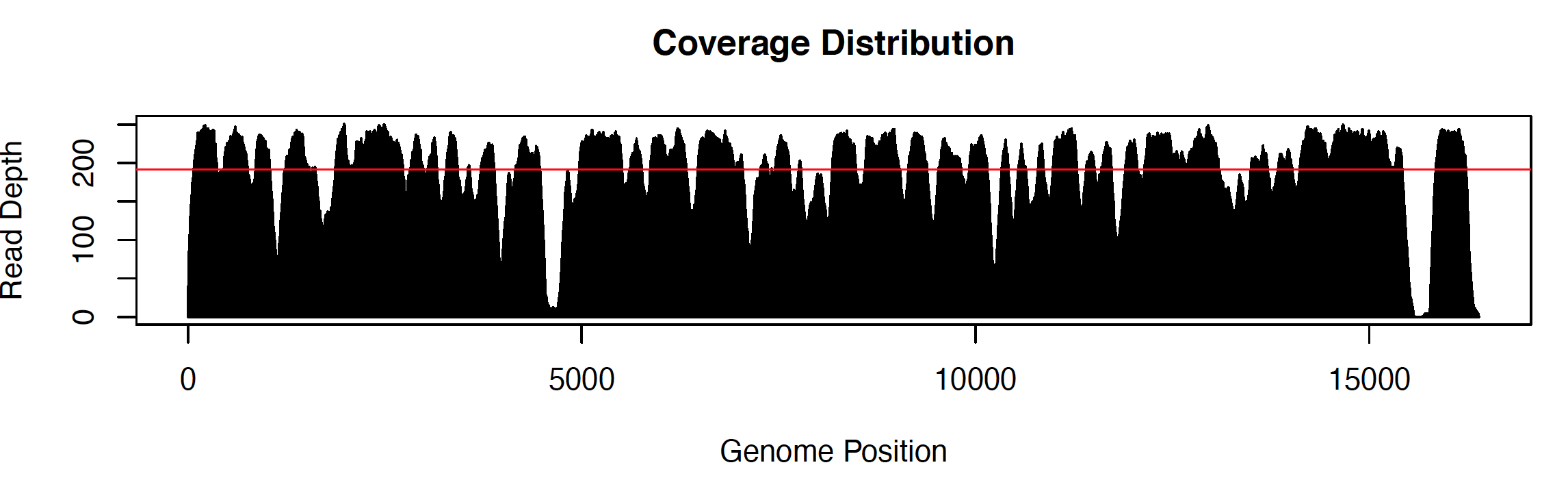
**

**3.11 KBA_20**

98.9% coverage, mean 25x

**
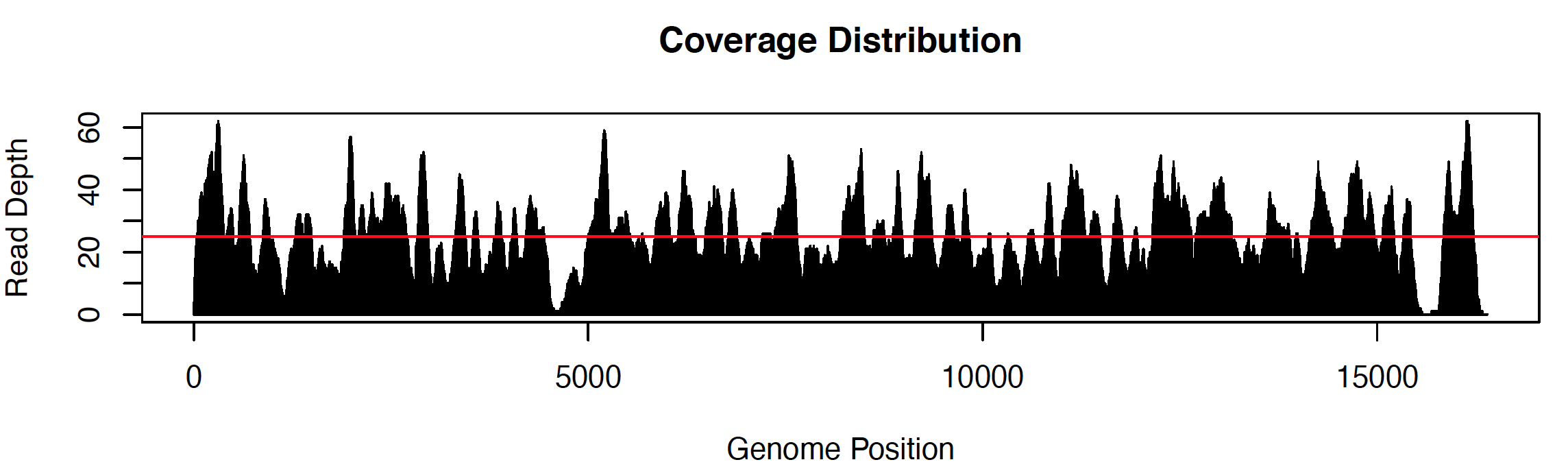
**

**3.12 KBB_22**

99.7% coverage, mean 137.4x

**
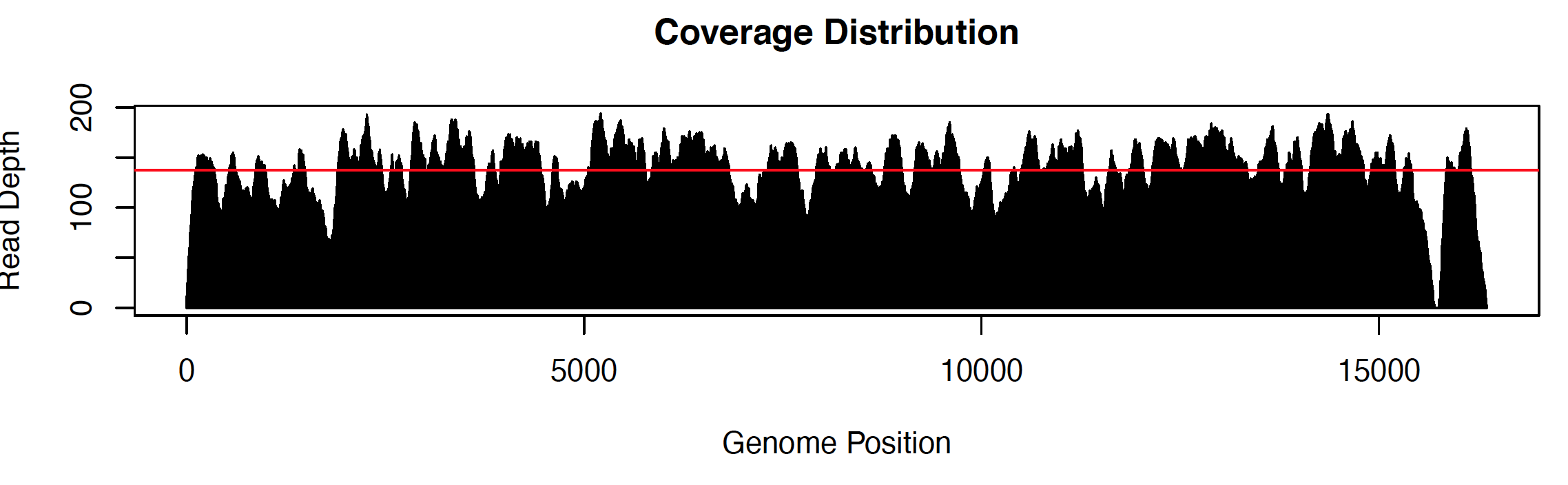
**

**3.13 KBB_25**

97.2% coverage, mean 10.3x


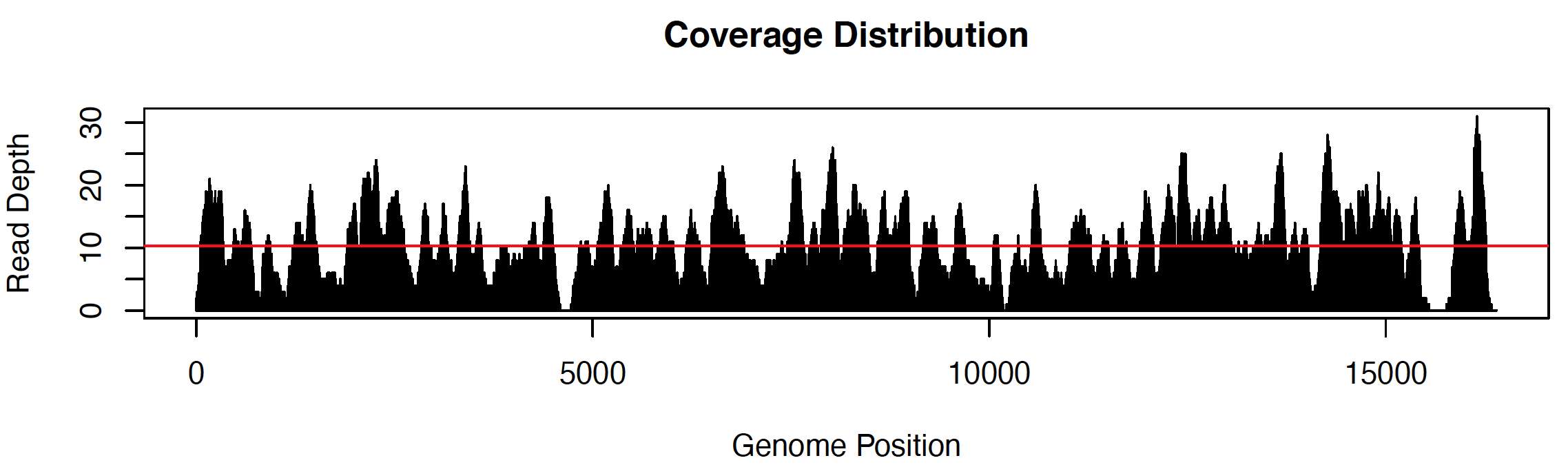


**Figures S4**

**4.1. Maximum likelihood tree showing relationship of the ancient buffalo from this study to other publicly available buffalo sequences from Genbank**

**
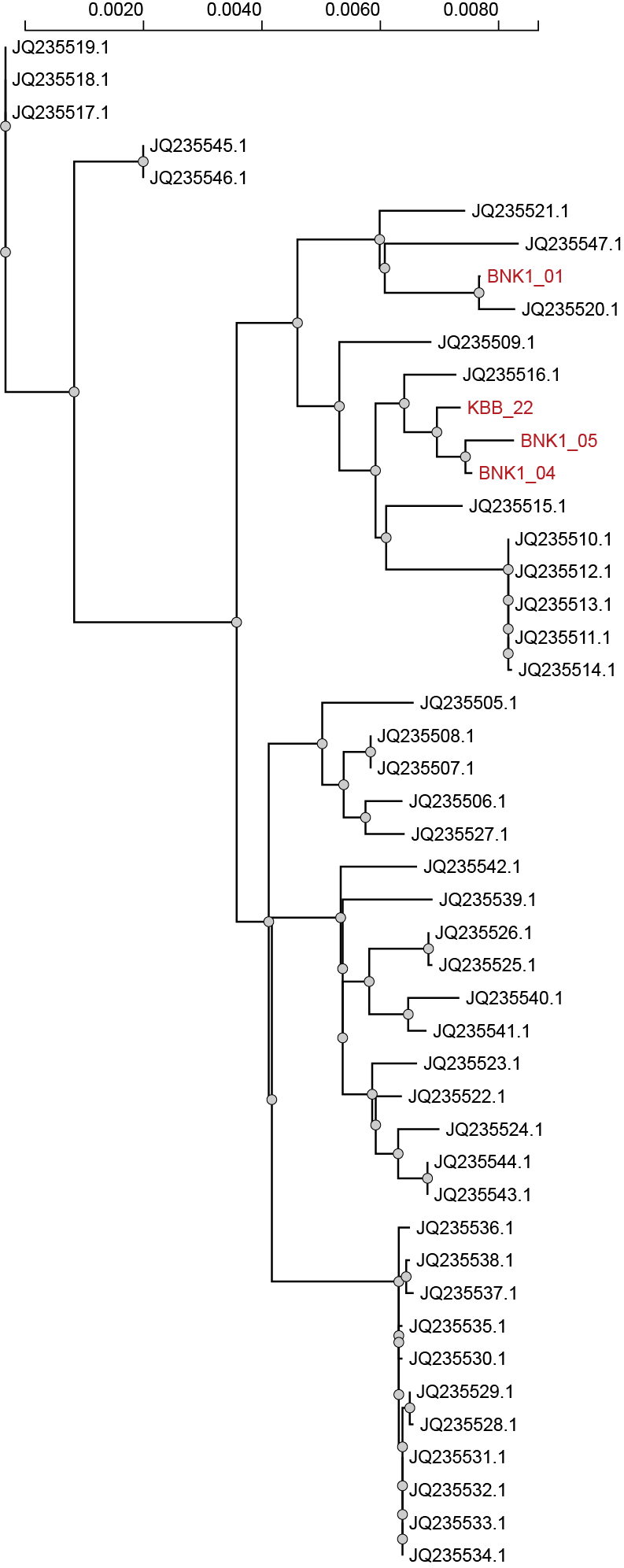
**

**4.2 Maximum likelihood tree showing relationship of the ancient eland and gemsbok from this study to other publicly available sequences from Genbank**

**
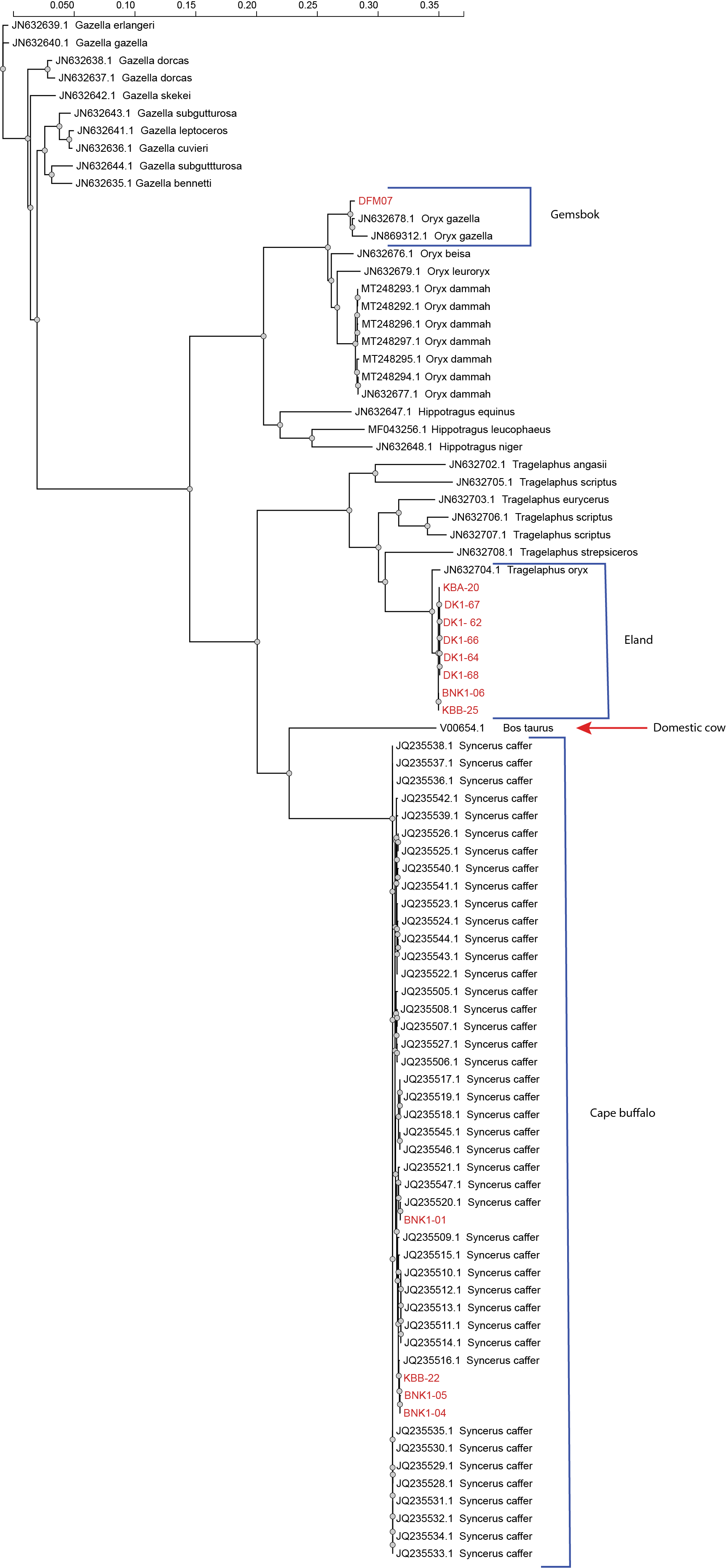
**
